# Assessment of Interleukin 16 Serum Levels and Skin Expression in Psoriasis Patients in Correlation with Clinical Severity of the Disease

**DOI:** 10.1371/journal.pone.0165577

**Published:** 2016-10-27

**Authors:** Dorota Purzycka-Bohdan, Aneta Szczerkowska-Dobosz, Monika Zablotna, Justyna Wierzbicka, Anna Piotrowska, Michal A. Zmijewski, Boguslaw Nedoszytko, Roman Nowicki

**Affiliations:** 1 Department of Dermatology, Venereology and Allergology, Medical University of Gdansk, Gdansk, Poland; 2 Department of Histology, Medical University of Gdansk, Gdansk, Poland; University of Alabama at Birmingham, UNITED STATES

## Abstract

Interleukin 16 (IL-16) has been described as a significant cytokine involved in the recruitment of CD4+ cells during inflammation; however, its potential role in psoriasis has not been defined. Our aim was to investigate the IL-16 serum levels and *IL-16* mRNA skin expression in psoriasis patients in correlation with disease severity and mRNA skin expression for *CD4*. Moreover, the IL-16 skin localization was assessed and the -295 T/C *IL-16* polymorphism was analyzed. For this exploratory, observational, and cross-sectional study, 97 unrelated patients with chronic plaque type psoriasis and 104 healthy controls were enrolled. IL-16 serum levels were significantly increased in patients compared with controls (*P* = 0.000022) and positively correlated with Psoriasis Area and Severity Index (*r* = 0.34, *P* = 0.0007), Body Surface Area (*r* = 0.34, *P* = 0.01) and were significantly higher in individuals with moderate to severe psoriasis (*P* = 0.0029). There was no significant correlation between IL-16 serum levels and Dermatology Quality of Life Index and no differences in genotype and allele frequencies for -295 T/C *IL-16* polymorphism. The expression of IL-16 (mRNA and protein) was elevated in the margin of psoriatic skin while statistically significant increase in IL-16 immunoreactivity, but not in mRNA level, was observed within plaques. Furthermore, the *IL-16* mRNA levels within psoriatic lesions positively correlated with the levels of *CD4* mRNA, but not with Psoriasis Area and Severity Index. In conclusion, our data revealed an association between circulating IL-16 and severity of psoriasis which indicates that this cytokine could serve as a potential marker of disease activity. However, further investigations are required.

## Introduction

Psoriasis is a chronic skin disease characterized by scaly red lesions as a consequence of abnormal proliferation of keratinocytes, cutaneous inflammation and skin vessel disturbances [1]. Many studies have shown that T lymphocytes play a significant role in the pathogenesis of psoriasis [2]. In 1994 Prinz et al. proved that T cell clones from psoriatic skin lesions can promote keratinocyte proliferation *in vitro* [3]. In other studies [4,5] authors observed that highly purified blood-derived CD4+ T cells induced psoriatic plaques when injected into symptomless skin engrafted onto severe combined immunodeficiency (SCID) mice.

Interleukin-16 (IL-16) was firstly described by Dr. David Center and Dr. William Cruikshank from Boston University [6]. It was originally known as lymphocyte chemoattractant factor (LCF), based on the initial observations for the induction of CD4+ T lymphocyte chemotaxis. Since then, IL-16 has been shown to recruit and activate many other cells expressing the CD4 molecule, including mast cells, eosinophils, and dendritic cells [7,8]. As opposed to most other precursor molecules, both the pro-molecular and the mature forms of IL-16 have been found to be biologically active [9]. In lymphocytes, the N-terminal domain of the precursor protein (pro-IL-16) translocates into the nucleus and functions as a transcriptional repressor with regulatory effects on cell cycle, whereas mature IL-16 (the cytoplasmic C-terminal domain) is secreted from the cell as a ligand for CD4 with chemoattractant, growth factor, and differentiation factor capabilities on a variety of hematopoietic cell types, which are involved in an inflammatory response [9]. Nevertheless, the biological function of IL-16 is not yet fully known.

The pleiotropic impact of IL-16 on immune system cells and its association with CD4+T cells suggest that this cytokine might be involved in the pathogenesis of psoriasis. There are only a few studies in literature concerning IL-16 in this disease. The aim of this study was to investigate whether the serum levels of IL-16 and *IL-16* mRNA (messenger RNA) skin expression correlate with clinical severity of psoriasis and mRNA skin expression for *CD4*. Moreover, genotype and allele frequencies for -295 T/C *IL-16* promoter gene polymorphism were analyzed.

## Material and Methods

### Patients

For this exploratory, observational, and cross-sectional study, 97 unrelated patients with chronic plaque type psoriasis admitted to the dermatology department and to the dermatology outpatient clinic were enrolled. Patients included (the age ≥ 18 years) had not received systemic treatment for psoriasis (cyclosporine, methotrexate, retinoids, corticosteroids, phototherapy) for the previous three months or topical anti-psoriatic therapy for the previous one week. Current or previous biological therapies constituted exclusion criteria. Individuals with other chronic skin disorders, psoriatic arthritis, patients on current immunosuppressive therapy, organ transplant recipients or individuals suffering from any other systemic inflammatory diseases or malignancy were excluded from the study participation.

### Controls

The control group consisted of 104 adult, healthy, unrelated subjects. Recruitment for the control group covered healthy volunteers (mainly blood donors) without psoriasis and free from other chronic inflammatory skin and systemic diseases. In all volunteers dermatological examination with the skin assessment was performed to exclude presence of possible skin lesions. Individuals with positive family history of psoriasis were also excluded.

All subjects were exclusively of Eastern Europe/Polish descent.

The study was approved by the Local Ethics Committee at the Medical University of Gdansk and performed in accordance with the Declaration of Helsinki. Written informed consent was obtained from all individual participants included in the study.

### Psoriasis severity assessment

In all patients, dermatological examination with psoriasis severity assessment using Psoriasis Area and Severity Index (PASI), Body Surface Area (BSA) and Dermatology Quality of Life Index (DLQI) was performed by the same dermatologist.

### IL-16 serum levels

In all patients and healthy individuals, the blood was drawn and afterwards serum levels of IL-16 protein were measured using Quantikine ELISA (enzyme-linked immunosorbent assay) test [USA&Canada R&D Systems, Inc., Minneapolis].

### *IL-16* mRNA levels in skin samples

For mRNA isolation and immunohistochemical analysis, the 4 mm in diameter biopsies from disease affected skin (psoriatic lesion) of the buttocks and additionally from potentially unaffected margin (marginal tissue in the distance of about 2cm from plaque) in volunteers from the psoriasis group, and healthy skin of the buttocks in volunteers from the control group were taken after obtaining written informed consent. This body area was chosen mainly due to its low exposure to the sun and low visibility of small scars. To avoid possible modification of the skin inflammation, the biopsies were taken only from patients of the study group who did not receive topical anti-psoriatic treatment in the skin region of buttocks for the previous four weeks [10,11].

The relative mRNA levels of analyzed genes (IL-16, CD4) were established by quantitative polymerase chain reaction (qPCR) in matching samples (psoriatic lesion and marginal skin) and control biopsies. Following mechanical homogenization of tissue samples, total RNA was isolated by the Total RNA Mini kit (A&A Biotechnology, Gdynia, Poland). The amount and purity of the RNA samples were determined spectrophotometrically (Epoch BioTek, Winooski, USA). One microgram of RNA was used for reverse transcription performed with a RevertAid™ First Strand cDNA Synthesis Kit (Thermo Scientific, Waltham, USA). The Real-Time PCR reaction was carried out using StepOnePlus™ Real-Time PCR System (Life Technologies-Applied Biosystems, Grand Island, NY, USA) with PCR Master Mix SYBR^®^ kit (A&A Biotechnology, Poland) according to the manufacturer's protocol. The expression of genes was normalized by comparative ΔΔ-*C*_t_ method, using *RPL37* as a housekeeping gene, followed by calibration (fold change) to normalized expression data of samples from control patients (ratio = 1).

### Immunostaining

Frozen skin samples (psoriatic skin and matching marginal biopsies, and healthy control samples) were sliced on a microtome into 10 μm thick sections followed by 10 minutes fixation in 4% paraformaldehyde in PBS pH 7.4. After washing (3 x 5 minutes in PBS), specimens were permeabilised for 5 minutes in 0.2% Triton X100 in PBS. Following the washing (3 x 5 minutes in PBS), the slides were incubated for 30 minutes at RT with 1% BSA in PBS. After blocking, primary antibody (rabbit polyclonal anti-IL-16 Sigma HPA018467) diluted in 1%BSA/PBS was applied and the specimens were incubated in humidified chamber at 4°C overnight. After indicated time, the primary antibody solution was decanted and the slides were washed three times in PBS, followed by incubation with secondary antibody (goat anti rabbit IgG Thermo Fisher Scientific A11008) solution in 1%BSA/PBS for 1 hour at RT in dark. Following the washing (3 x 5 minutes in PBS), the sections were counterstained with 4’,6’-diamidinio-2-phenylindole (DAPI). Microscopy was performed with a Nikon Eclipse E800, measurements of fluorescence intensity were done with Image J Software.

### *IL-16* genotyping

Genomic DNA was extracted from EDTA blood by standard techniques using Blood Mini kit (A&A Biotechnology, Poland) according to the manufacturer’s instruction.

In all patients and control subjects the -295 T/C (rs4778889) promoter gene polymorphism of IL-16 was analyzed using polymerase chain reaction–restriction fragment length polymorphism (PCR-RFLP) method as previously described by Romani et al. [12]. Amplification was carried out in 25 μl volume containing 200 ng genomic DNA, 0.1 μM of each primer, 200 μM of each dNTP, 2.5 mM of MgCl and 1 U of REDTaq polymerase (Sigma-Aldrich, USA). Amplification was conducted in thermal cycler Mastercycler Gradient (Eppendorf, Germany) under the following conditions: one cycle of initial denaturation at 94°C for 4 min followed by 35 cycles of denaturation at 94°C for 45 s, annealing at 60°C for 45 s, extension at 72°C for 45 s then a final extension cycle at 72°C for 7 min. Digested with AhdI enzyme PCR products were separated and analyzed on 3% low melting temperature agarose gel (Agarose LM, Laboratorios Conda S.A., Spain).

### Statistical analysis

Statistical analyses of IL-16 serum levels and *IL-16* polymorphisms were performed with Statistica 10.0 software package (StatSoft, Inc., 2011). The Mann-Whitney U-test was used to compare the median values, and the correlation was determined using mean Spearman coefficient values. The χ^2^ analysis was used to compare the observed number of genotypes with that expected for a population in a Hardy-Weinberg equilibrium. The χ^2^ analysis was employed to test the significance of the differences in the observed alleles and genotypes between groups. The Kruskal-Wallis test was applied to compare IL-16 serum levels depending on genotypes. Real-Time PCR data were analyzed with the Student's t-test (for two groups) or one-way analysis of variance and appropriate post hoc test (the ANOVA Kruskal-Wallis test for comparison of several groups) using GraphPad Prism v6.03 (GraphPad Software, San Diego, CA, USA). The data are presented as median value with range (min-max). Statistical analysis of immunostaining of skin samples based on measurements of fluorescence intensities was conducted. From 5 to 7 random fields covering epidermis for each specimen were analyzed using micrographs taken under 200x magnification. Statistical analysis was performed using GraphPad Prism v6.03 (GraphPad Software, San Diego, CA, USA). The data were subjected to Student’s t-test (for two groups). A *P*-value < 0.05 was considered statistically significant.

## Results

In the study group, the mean PASI was 17.2; moderate to severe psoriasis with PASI>10 was observed in 75 patients (77.3%). There were 71 patients with early-onset and 26 with late-onset psoriasis.

The characteristics of study participants are presented in Table 1.

**Table 1:** Characteristics of the study group vs. the control group.

	Psoriasis group	Early-onset psoriasis (< age 40 years)	Late-onset psoriasis (> or = age 40 years)	Psoriasis patients with biopsies	Control group	Controls with biopsy
**Total number of subjects, n (%)**	97 (mild n = 22; moderate to severe n = 75)	71 (73.2)	26 (26.8)	25	104	21
**Males, n (%)**	57 (58.8)	43 (60.6)	14 (53.8)	14 (56)	39 (37.5)	10 (47.6)
**Females, n (%)**	40 (41.2)	28 (39.4)	12 (46.2)	11 (44)	65 (62.5)	11 (52.4)
**Age at enrolment (years), Mean (SD)**	48.5 (15.9)	44.0 (16.1)	60.3 (7.1)	49.9 (16.7)	41.6 (16.3)	47 (15.7)
**Age at onset of symptoms (years), Mean (SD)**	27.3 (15.9)	19.4 (8.1)	50.9 (8.1)	29.0 (16.5)	-	-
**PASI, Mean (SD)**	17.2 (9.2)	17.4 (9.3)	16.6 (9.0)	15.5 (6.4)	-	-
**Positive family history, n (%)**	49 (50.5)	38 (54.3)	11 (40.7)	16 (64)	0	0

n-number of participants; SD- standard deviation; PASI—Psoriasis Area and Severity Index.

**Fig 1:**
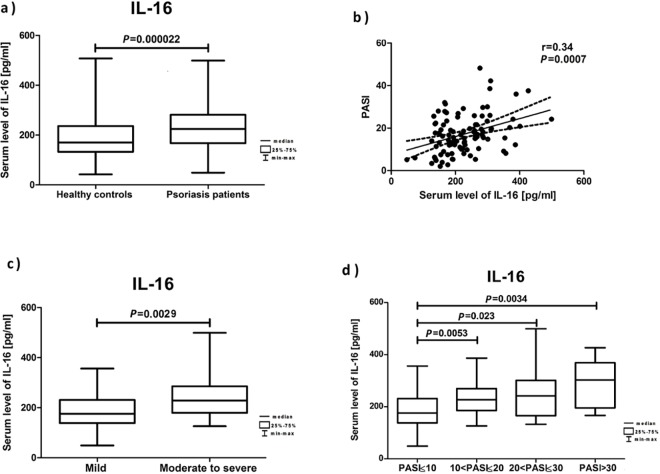
Analysis of IL-16 levels in serum. The figure presents the cytokine serum levels: (a) in healthy controls and psoriasis patients; (b) in correlation with PASI; (c) in patients with mild (PASI≤ 10) vs. moderate to severe (PASI>10) psoriasis; (d) based on PASI.

Serum levels of IL-16 were significantly increased in patients with psoriasis compared with unaffected subjects (Fig 1A). Moreover, IL-16 serum levels positively correlated with PASI (Fig 1B) and BSA (*r* = 0.34, *P* = 0.01) and were significantly higher in the group of patients with moderate to severe psoriasis (Fig 1C and 1D). There were no significant statistical differences between the interleukin serum levels in patients with mild psoriasis vs. control subjects (*P* = 0.77), early-onset vs. late-onset psoriasis (*P* = 0.07) and no significant correlation between IL-16 serum levels and the quality of life assessed by DLQI (*r* = 0.03; *P* = 0.83).

The detailed results of IL-16 serum levels are presented in Table 2.

**Table 2:** IL-16 serum levels in the study participants.

	n	Mean (pg/ml)	SD (pg/ml)	Q25 (pg/ml)	Median (pg/ml)	Q75 (pg/ml)
**Psoriasis group**	97	229.98	79.90	168.37	224.50	280.41
**Control group**	104	186.88	75.91	133.07	170.29	235.95
**Early-onset psoriasis**	71	238.89	85.16	168.37	228.50	285.56
**Late-onset psoriasis**	26	205.64	58.05	166.35	191.31	261.30
**Mild psoriasis**	22	187.55	74.87	139.66	176.02	234.57
**Moderate to severe psoriasis**	75	242.42	77.46	179.65	228.50	285.56
**PASI< = 10**	22	187.55	74.87	139.66	176.02	234.57
**10<PASI< = 20**	45	230.24	58.73	185.96	227.20	267.57
**20<PASI< = 30**	23	251.21	97.06	166.70	258.78	299.50
**PASI>30**	7	291.88	99.47	168.37	307.00	388.78

n-number of participants; SD-standard deviation; Q25-lower quartile; Q75-upper quartile; PASI-Psoriasis Area and Severity Index.

**Fig 2:**
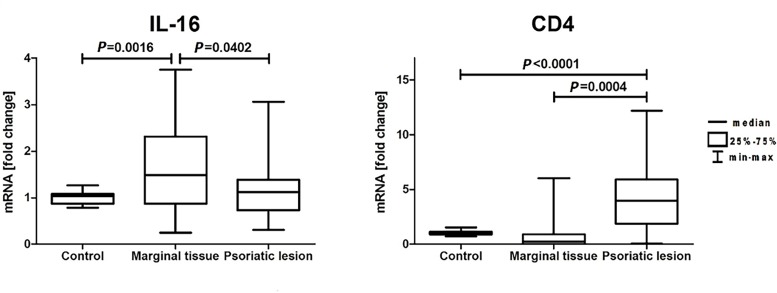
Relative mRNA levels of *IL-16* and *CD4* genes in skin samples.

**Fig 3:**
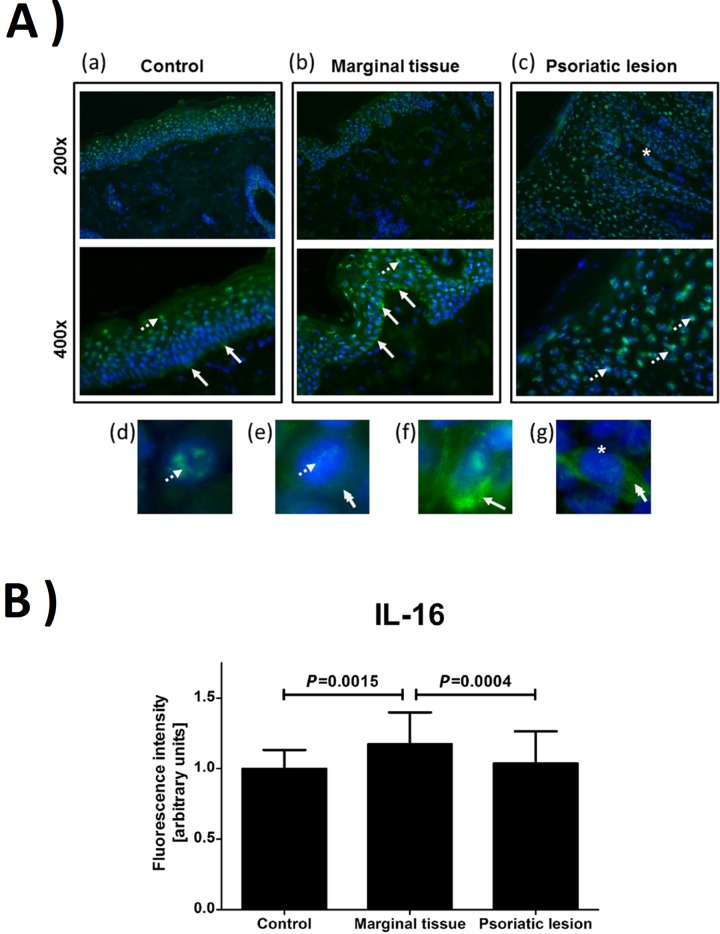
Immunostaining of IL-16. (A) Immunodetection of IL-16 immunoreactivity (green) in skin biopsies—control, marginal tissue and psoriatic lesion (a,b,c). The examples of IL-16 nuclear/perinuclear localization (arrows with dotted line), cytoplasmic (double head arrow), cytoplasmic in keratinocytes close to the basement membrane (arrow) and cytoplasmic in cells of dermal papilla (star) are marked on microphotographs. Panels d,e,f and g present IL-16 immunoreactivity within single cells. The nuclei were stained with blue. (B) The relative change in the intensity of immunofluorescence characteristic for IL-16 was quantified in the epidermis.

**Fig 4:**
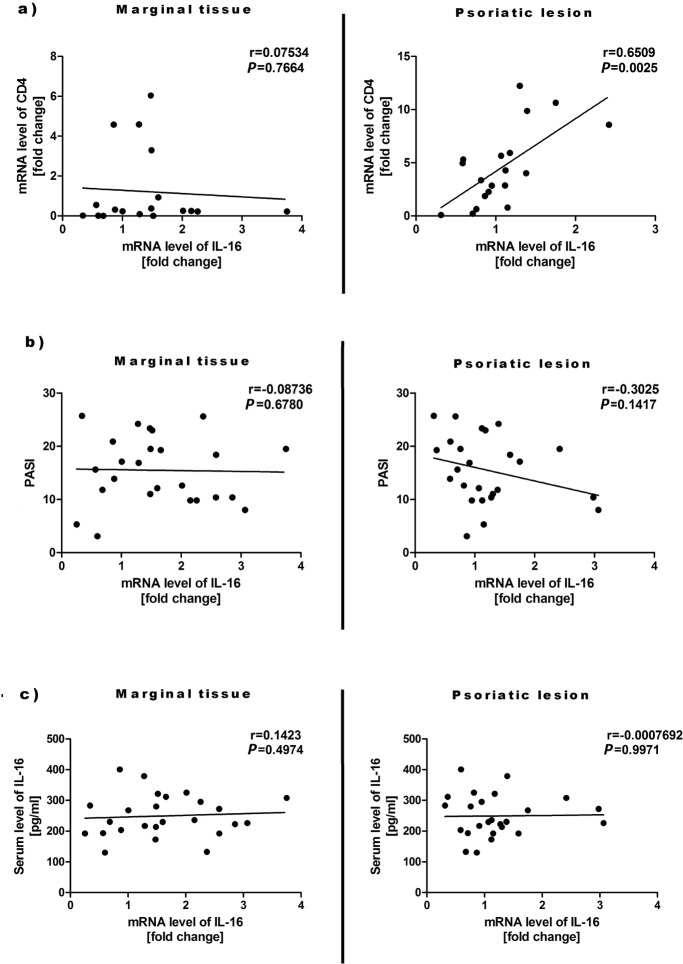
The *IL-16* mRNA levels in skin samples in correlation with the mRNA levels for *CD4* (a), PASI (b) and IL-16 serum levels (c).

The skin biopsy of psoriatic lesion and potentially unaffected margin in 25 volunteers from the psoriasis group and the biopsy of healthy skin in 21 individuals from the control group were taken. It was shown that the expression of mRNA for *IL-16* was mainly elevated in the margin of psoriatic skin (Fig 2). Similar effect was observed by immunodetection of specific IL-16 epitops in skin biopsies (Fig 3A and 3B). In addition, statistically significant increase in IL-16 immunoreactivity (Fig 3A and 3B), but not in mRNA level (Fig 2), was observed in psoriatic lesions. Interestingly, it seems that the IL-16 immunoreactivity is preferentially localized in the nucleus or perinuclear area in lesional skin, while more cytoplasmic IL-16 immunoreactivity was observed in the marginal tissue and skin of healthy controls (Fig 3A). Finally, elevated level of *CD4* mRNA was detected in psoriatic skin (Fig 2) and it positively correlated with increased level of mRNA for *IL-16* (Fig 4A). The *IL-16* mRNA levels in the margin of psoriatic lesions as well as within plaques did not correlate with PASI and IL-16 serum levels (Fig 4B and 4C).

There were no statistically significant differences in genotype (*P* = 0.97) and allele (*P* = 0.84) frequencies for -295 T/C *IL-16* promoter gene polymorphism in patients with psoriasis and control subjects as well as in subgroups of early-onset and late-onset psoriasis (*P* = 0.84) and mild vs. moderate to severe psoriasis (*P* = 0.73). Moreover, we did not observe differences in IL-16 serum levels based on genotypes (*P* = 0.59 in patients, *P* = 0.12 in controls).

## Discussion

The pleiotropic mechanism of IL-16 may suggest its role in the pathogenesis of cutaneous disorders closely associated with infiltration of CD4+ T-cells. Up till now, this cytokine has been studied in atopic dermatitis (AD) [13,14], systemic lupus erythematosus (SLE) [15], pemphigoid [16] and cutaneous T-cell lymphomas [17]. Increased IL-16 serum levels, which additionally correlated with disease severity, were proved in patients with these disorders [13,15,17]. In addition, the authors suggested IL-16 as a marker of AD, SLE and Sezary's syndrome activity. To our knowledge, there are three studies assessing IL-16 in serum of psoriasis patients. Nagy et al. [18] and Zheng et al. [19], who investigated the association between IL-16 and the degree of sensitization in AD, observed significantly higher levels of IL-16 in the sera of patients with AD, compared to healthy individuals and patients with chronic plaque type psoriasis. Similarly, Masuda et al. [20] noticed lower IL-16 serum levels in patients with psoriasis than with AD, although significantly higher than in control subjects. The authors suggested the difference between psoriasis and AD in a Th1/Th2 balance as one of the reasons for the disparity in IL-16 serum levels [20]. However, they stated that the literature does not point to Th2 cells as the preferential source of this cytokine. Nevertheless, in these three studies the psoriasis groups were relatively small (12–20 patients) and young (an average age of 19.6–28.2 years). In our research, serum levels of IL-16 were significantly elevated in the group of 97 patients (mean age 48.5 years) compared to controls. Moreover, we also revealed the progressive increase in IL-16 serum levels with the increase in severity of the disease, documented by the positive correlation between serum IL-16 and PASI as well as BSA. Circulating biomarkers have been extensively studied in psoriasis because of the easy accessibility to patients’ peripheral blood samples, but this correlation has not been investigated so far. The potential limitation for using IL-16 serum levels as a marker of disease activity would be that the level of this cytokine can be increased in the course of several disorders with acute or chronic inflammatory reactions. For example, the elevated levels of IL-16 in serum were reported in diabetes, metabolic syndrome and cardiovascular disease which are often associated with psoriasis [21,22].

Wagner et al. [23] suggested IL-16 as a potential predictive marker for PASI75 response (specificity 63%, sensitivity 76%) measured in psoriatic arthritis patients treated with golimumab. In our study, patients with psoriatic arthritis and individuals who received biological therapies were excluded to provide a clearance of psoriasis group and avoid potential influence of these factors on IL-16 levels.

Based on biological properties of this cytokine and the parallel activation of the interleukin-2/CD25 pathway, Blaschke et al. [24] postulated that IL-16 might be involved in the stimulation and recruitment of CD4+ lymphocytes in mycosis fungoides lesions and therefore contribute to the perpetuation of skin inflammation. Moreover, upregulation of *IL-16* mRNA was reported to be associated with increased numbers of CD4+ cells in acute AD skin lesions [14]. Furthermore, Skundric et al. [25] demonstrated effectiveness of anti-IL-16 therapy in reducing CD4+ T-cell infiltration of the central nervous system in experimental autoimmune encephalomyelitis in mice. Interestingly, in this study, although there was no correlation between level of *CD4* mRNA in the skin lesions and IL-16 in the serum or PASI score (not shown), the considerably elevated level of IL-16 in the margin of psoriatic skin and shown positive correlation between levels of mRNA for *IL-16* and *CD4* within plaques suggest potential involvement of IL-16 in expansion of psoriatic lesions. According to previous studies [14,24] we also observed low but detectable expression of epidermal *IL-16* mRNA in normal skin. Moreover, it seems that disperse cytoplasmic immunoreactivity for IL-16 prevails in the skin of healthy donors, while more condense, perinuclear and nuclear aggregates of IL-16 are characteristic for psoriatic epidermal keratinocytes. It was suggested that nuclear localization of pro-IL-16 (full length protein) leads to inhibition of immortalized human T lymphocyte cells (Jurcat) through the G0/G1 cell arrest [26,27]. It is still unknown whether similar mechanism functions in keratinocytes, however, observed perinuclear/nuclear localization of IL-16 immunoreactivity in psoriatic skin may suggest adverse mechanism of pro-IL-16 activity in keratinocytes or perinuclear accumulation of active form of IL-16. Interestingly, elevated immunoreactivity for IL-16 was observed in basal layer of keratinocytes in close proximity to basement membrane. It may suggest active exchange of this cytokine between epidermis and dermis, which may result in chemoattraction of CD4 expressing cells and/or modulation of keratinocytes proliferation, resulting in progression of psoriatic lesions. However, the detailed mechanism of IL-16 impact on the development and progression of psoriasis requires clarification due to its potential cooperation and interdependencies with other proinflammatory cytokines and immune system cells.

For this research we selected one previously validated common single nucleotide polymorphism (SNP) in the *IL-16* gene, rs4778889 T/C, located at 295 bp upstream from the start site of transcription that is associated with an expression level of IL-16 protein [28,29]. Several studies of autoimmune and inflammatory disorders indicate an association between this polymorphism and an increased risk of diseases. Xue et al. [30] examined the rs4778889 T/C variants among patients with SLE and healthy controls and found a significant association between this SNP and susceptibility to SLE in a Chinese population. Azimzadeh et al. [28] suggested influence of rs4778889 T/C polymorphism on the altered risk of colorectal cancer. This study is the first report on *IL-16* gene polymorphism among psoriasis patients. Nevertheless, we did not observe differences in genotype and allele frequencies for -295 T/C *IL-16* gene polymorphism in this population. Furthermore, we did not notice differences in IL-16 serum levels based on genotypes.

## Conclusions

This preliminary study provides the description of *IL-16* presence in psoriatic skin. The results demonstrate that upregulation of *IL-16* mRNA expression within psoriatic plaques is associated with increased number of CD4+ cells, however, the highest *IL-16* expression was found within apparently uninvolved skin around the lesions. Moreover, the research revealed significant positive correlation between IL-16 levels in serum and clinical severity of psoriasis which indicates that this cytokine could serve as a potential marker of disease activity. Nevertheless, further clinical observations and subsequent studies are needed to assess if individuals with mild psoriasis and higher level of circulating IL-16 are more predisposed to disease progression and whether the levels of IL-16 previous to treatment onset correlate to response to anti-psoriatic therapy.
